# Abnormal microscale neuronal connectivity triggered by a proprioceptive stimulus in dystonia

**DOI:** 10.1038/s41598-020-77533-w

**Published:** 2020-11-27

**Authors:** Dimitris F. Sakellariou, Sofia Dall’Orso, Etienne Burdet, Jean-Pierre Lin, Mark P. Richardson, Verity M. McClelland

**Affiliations:** 1 Department of Basic and Clinical Neuroscience, Institute of Psychiatry, Psychology and Neuroscience, King’s College London, London, SE5 9RX UK; 2 Machine Learning & Artificial Intelligence Solutions Global Unit, Real World Solutions, IQVIA, London, N1 9JY UK; 3 Department of Biomedical Engineering and Human Robotics, Imperial College London, London, SW7 2AZ UK; 4 Children’s Neurosciences Department, Evelina London Children’s Hospital, Guy’s and St Thomas’ NHS Foundation Trust, London, SE1 7EH UK

**Keywords:** Dystonia, Statistics, Electrical and electronic engineering

## Abstract

We investigated modulation of functional neuronal connectivity by a proprioceptive stimulus in sixteen young people with dystonia and eight controls. A robotic wrist interface delivered controlled passive wrist extension movements, the onset of which was synchronised with scalp EEG recordings. Data were segmented into epochs around the stimulus and up to 160 epochs per subject were averaged to produce a Stretch Evoked Potential (StretchEP). Event-related network dynamics were estimated using a methodology that features Wavelet Transform Coherency (WTC). Global Microscale Nodal Strength (GMNS) was introduced to estimate overall engagement of areas into short-lived networks related to the StretchEP, and Global Connectedness (GC) estimated the spatial extent of the StretchEP networks. Dynamic Connectivity Maps showed a striking difference between dystonia and controls, with particularly strong theta band event-related connectivity in dystonia. GC also showed a trend towards higher values in dystonia than controls. In summary, we demonstrate the feasibility of this method to investigate event-related neuronal connectivity in relation to a proprioceptive stimulus in a paediatric patient population. Young people with dystonia show an exaggerated network response to a proprioceptive stimulus, displaying both excessive theta-band synchronisation across the sensorimotor network and widespread engagement of cortical regions in the activated network.

## Introduction

The underlying mechanisms of dystonia remain elusive. The basal ganglia are implicated in the pathophysiology because lesions in this area may produce dystonia, but in many forms of dystonia structural damage to the basal ganglia is not seen and neuroimaging is often considered normal^1^. Neurophysiological and functional imaging studies demonstrate increased receptive fields for sensory representation of clinically affected areas^2^ and impaired gating of afferent information^3,4^, providing strong evidence that dystonia is a disorder of impaired sensorimotor processing. Dystonia is now widely accepted as a network disorder with many brain regions implicated, including basal ganglia, sensorimotor cortex and cerebellum^1^. Considering dystonia as a network disorder, a structural or developmental abnormality of one or more nodes of the network and/or abnormal communication between nodes may result in clinical features of dystonia. Abnormal connectivity in both the cortico-basal ganglia and the cortico-cerebellar networks has been demonstrated in writer’s cramp using fMRI^5,6^ and a recent “lesion network mapping” study identified the cerebellum and somatosensory cortex as key regions showing abnormal connectivity within the sensorimotor network in cervical dystonia^7^.

Pathophysiological mechanisms that have been demonstrated in dystonia and which may act diffusely across the sensorimotor network include reduced inhibition at multiple levels of the nervous system (cortex, brainstem and spinal cord)^8^ and exaggerated plasticity^9,10^. In addition, electrophysiological recordings in dystonia reveal pathologically enhanced low frequency (4–8 Hz) oscillatory activity in the basal ganglia–cortical network^11^. Suppression of this activity by Deep Brain Stimulation (DBS) of the Globus Pallidus internus (GPi) is associated with clinical improvement in adults with idiopathic/genetic dystonia^12^. The transmission of this low frequency activity into the peripheral motor system is suggested by coherence between pallidal low frequency local field potentials and dystonic EMG^13^ and by the presence of abnormal low frequency intermuscular coherence in several genetic dystonias^14,15^ and in acquired dystonias^16^. Moreover the magnitude of this exaggerated low frequency intermuscular coherence correlates with dystonia severity^16^ and is reduced by Deep Brain Stimulation^17^.

Recent work has indicated that event-related changes in cortical oscillatory activity and stimulus induced modulation of cortico-muscular coherence are abnormal in dystonia^16^. In light of these observations and the growing evidence of abnormal neuronal connectivity in dystonia, it is pertinent to examine how functional connectivity within the sensorimotor network is modulated by a sensory stimulus. The current study investigates dynamic changes in connectivity over the micro-scale of time (~ msec-sec), as triggered by a proprioceptive stimulus. We report preliminary data to support the application of this methodology in a paediatric patient population and demonstrate striking abnormalities in network organisation in dystonia, mediated via low frequency oscillatory activity.

## Results

**Figure 1:**
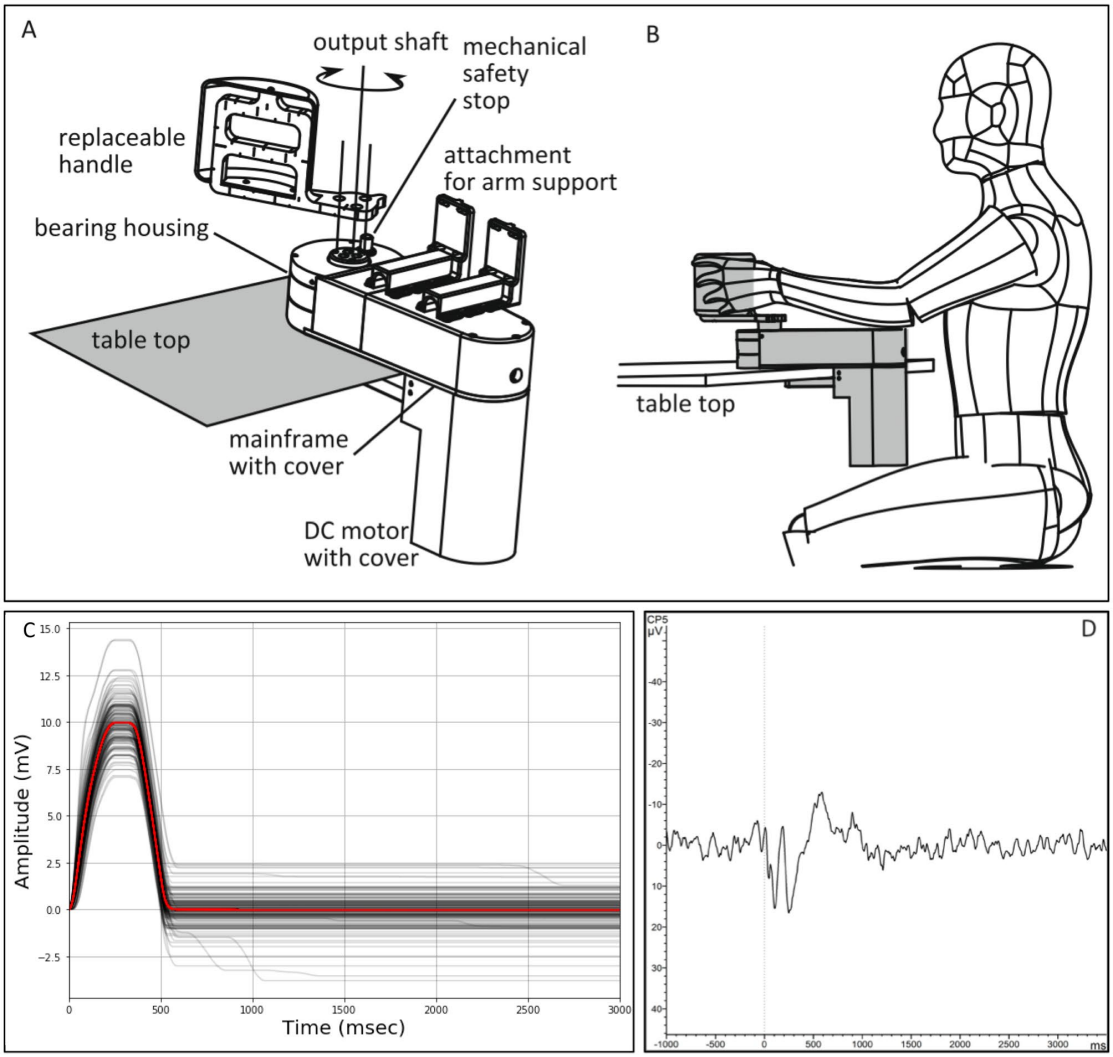
Top row: portable Hi5 interface for human motor control studies. (**A**) Design overview: the interface can be used with various handles and end effectors. (**B**) User interacting with Hi5 attached to a table-top. From Wilhelm et al.^35^. Line drawings kindly provided by Ildar Farkhatdinov. Bottom row: single subject data. (**C**) Movement profile of wrist extension in degrees from neutral position over time (ms). Each grey line shows the movement profile for an individual data epoch with the red line showing the mean. Produced with Python 3.7.2, matplotlib 3.1.0. (**D**) Cortical evoked potential recorded over contralateral sensorimotor cortex (in this case over CP5 electrode during right wrist movement). Figure shows average of 137 epochs, processed using BrainVision Analyser (Version 2.2; https://www.brainproducts.com).

Clinical details of the patients are shown in Table 1. Mean age was comparable between groups [Controls mean 10.9 years (95% CI 8.30–13.51), Dystonia mean 12.3 years (95% CI 10.09–14.56); independent samples t test (2-tailed) t = -0.841 p = 0.409]. Number of accepted epochs was also similar between groups (Controls mean 99.4, Dystonia mean 100). Mean wrist excursion was comparable between groups: Controls 9.68 degrees (95% CI 8.67–10.70), Dystonia 10.35 degrees (95% CI 9.18–11.52), independent samples t test: t = − 0.802 p = 0.431. The passive wrist extension evoked a clear and consistent potential over the contralateral sensorimotor cortex in each participant (Fig. 1D), maximal at C3, CP1 and CP5. The morphology of the response was comparable to previously published Stretch EPs^18^.

**Table 1:** Details of patients with dystonia.

Subject number	Age	Dystonia classification	Aetiology of dystonia	Phenotype	Dominant hand	BFMDRS-m	DBS	Global microscale nodal strength
1	8	Isolated genetic	DYT1	Generalised dystonia, frequent falls	R	57	N	0.0003
2	9	Isolated genetic	DYT1	Generalised dystonia	R	47	N	0.0087
3	10	Isolated genetic	DYT1	Generalised dystonia: initial craniocervical onset involving mouth opening and retrocollis followed by gait disturbance	R	38	Y	0.0203
4	11	Isolated genetic	DYT1	Generalised dystonia	R	52	N	0.0015
5	11	Isolated genetic	DYT1	Generalised dystonia	R		N	0.008
6	12	Isolated genetic	DYT11	Generalised dystonia with myoclonus	R	34.5	N	0.0110
7	16	Isolated genetic	DYT11	Generalised dystonia with myoclonus	R	20	Y	0.0033
8	7	Isolated genetic	KMT2B	Generalised movement disorder with both sustained dystonic postures and more hyperkinetic movments	R	46.5	N	0.0338
9	13	Isolated genetic	KMT2B	Generalised dystonia with dystonic tremor and possible Parkinsonian features	R	72	Y	0.0000
10	15	Isolated genetic	KMT2B	Generalised dystonia. Started in lower limbs, then arms and hand and progressing to involve jaw and speech	R	66	N	0.0033
11	20	Isolated genetic	KMT2B	Generalised dystonic choreoathetosis with possible myoclonic elements	R	72	Y	0.0059
12	11	Idiopathic	Idiopathic	Generalised but predominantly lower limb dystonia	R	24	N	0.0081
13	5	Acquired	Cerebral palsy due to term HIE	Generalised dystonia affecting predominantly left arm and both legs, with typical action dystonia	R	60	N	0.1358
14	17	Acquired	Cerebral palsy due to term HIE	Generalised dystonia with action specific dystonic tremor more marked with manual activities than gross motor skills. Dystonic dysarthria	L	27	Y	0.0236
15	7	Acquired	Cerebral palsy due to prematurity	Mixed movement disorder with features of dystonia and chorea	L		N	0.01
16	15	Acquired	Cerebral palsy due to prematurity	Generalised movement disorder with mixed dystonia and spasticity. Lower limbs most affected	L		N	0.0032

*HIE* hypoxic ischaemic encephalopathy, *BFMDRS-m* Burke–Fahn–Marsden Dystonia Rating Scale motor score, *DBS* deep brain stimulation.

### Global microscale nodal strength in theta band networks

**Figure 2:**
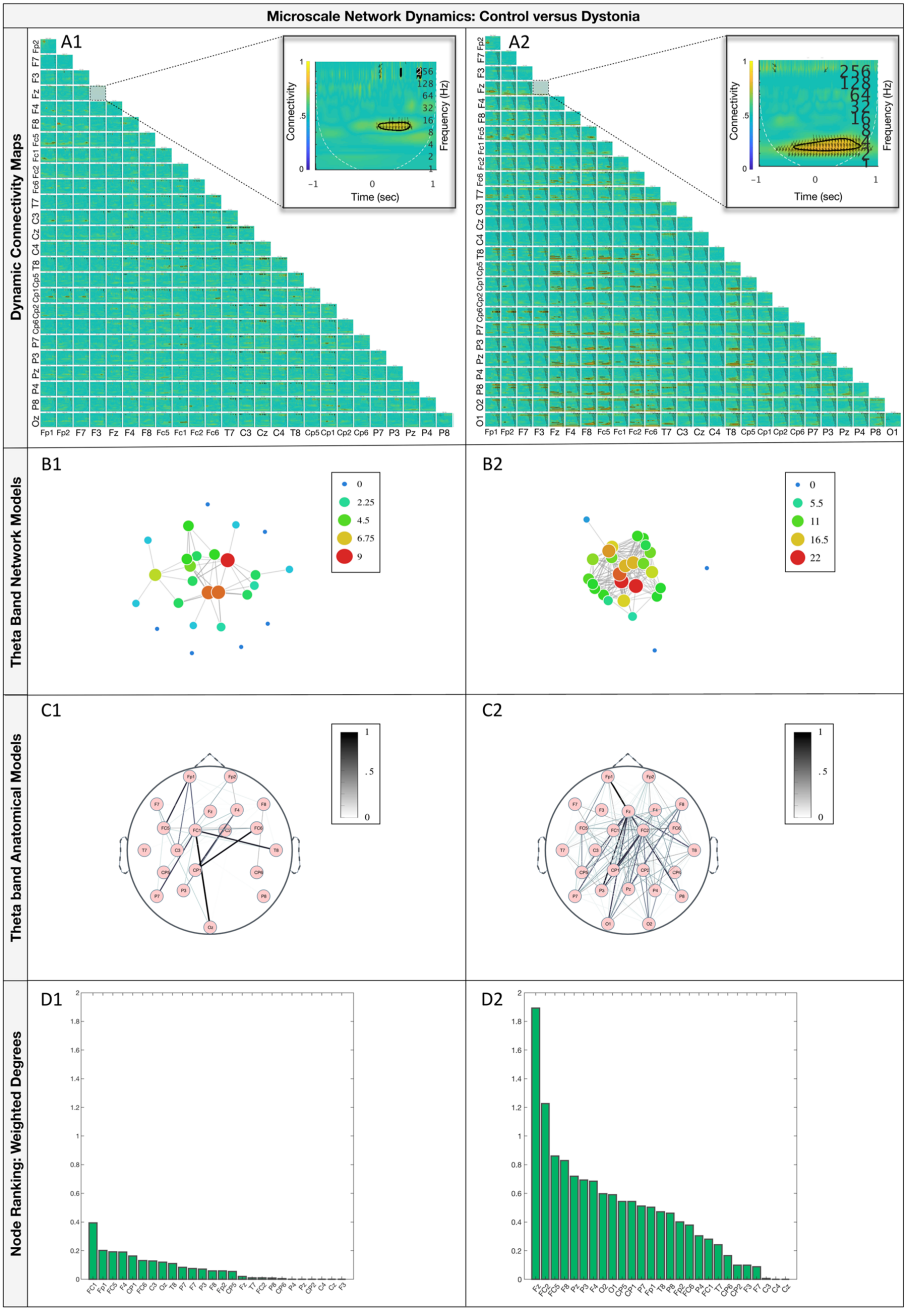
Network comparisons between example control (left) and patient (right) subjects, using data from two individual subjects, each representative of their respective subject group. Top row: subject-specific dynamic connectivity maps showing synchronisation values between all possible pairs of EEG electrodes for a typical control (**A1**) and participant with dystonia (**A2**). Electrodes are shown in X–Y axes and connection strength between x–y pairs of electrodes is indicated in cold (low) and warm (high) colours. The connectivity for a pair of electrodes is estimated over the time and frequency domains (x and y axis of subgraphs respectively) allowing for the characterisation of microscale network dynamics around an EEG event (t = 0 s), here the StretchEP. Arrows indicate the relative lag of significant connectivities for each pair of electrodes (right-horizontal: Δφ = 0, upward-vertical: Δφ = π/2, etc.). Upward and downward direction of arrows indicate flow of information from the electrode on the Y-axis towards the one in X-axis and vice versa. An expanded section is shown for an example electrode pair (Fz–F3) in each subject. Differences between the control and patient subjects in the connectivity values inside the subgraph boxes are evident and are further quantified in the network representations, in the second row. Produced with MATLAB 2018b and Neurocraft 1.0.0. Second row: network simulations. Theta-band connections of the peri-SEP networks are depicted in grey edges. The degrees (i.e. number of connections) for each EEG area/electrode are expressed in blue (least connections) to red (most connections). The positioning of the nodes was determined according to force-directed placement for undirected graphs, to reflect centrality features of the system^48^. The network simulations for the control (**B1**) and participant with dystonia (**B2**) reveal organisational dissimilarities that reflect differences in the number of areas that do not get involved in the network and more importantly significant differences in weighted degrees, as shown analytically in the bottom row. Produced with MATLAB 2018b and d3.js v5.16.0. Third row: anatomical representation of network patterns in the EEG space (**C1** control, **C2** participant with dystonia). Connection strengths are represented in thickness of links between areas and are scaled per subject from zero to one. EEG areas absent from the functional networks are not displayed in the graphs. Produced with “Easy Plot EEG Brain Network” (https://www.mathworks.com/matlabcentral/fileexchange/57372-easy-plot-eeg-brain-network-matlab). Bottom row: nodal ranking according to weighted degrees i.e. sum of connectivity values across all existing connections (**D1** control, **D2** participant with dystonia). Produced with MATLAB 2018b and Neurocraft 1.0.0.

Subject-specific dynamic connectivity maps showed a striking difference between individuals with dystonia compared with healthy controls (Fig. 2a) with the 4–8 Hz theta band standing out as showing significant and particularly strong event related connectivity in individuals with dystonia. The subsequent analysis was therefore focussed primarily on the theta band.

Although other connectivity activations at very high frequencies (> 120 Hz) can be visualised in the connectivity maps, the nature of these is uncertain. The experiment and analysis were not designed to investigate this range of activity and it is not considered further in this report.

**Figure 3:**
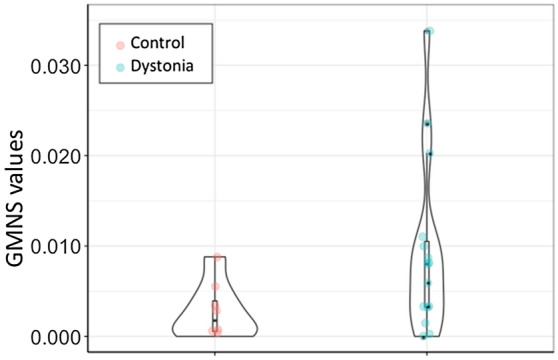
Global microscale nodal strength (GMNS) group differences. Split violin plots with inverted kernel densities and data points, exhibiting significant differences between the control and patient groups. Patients with DBS are denoted with asterisks. Produced with R 3.6.0 and ggplot 3.3.2.

The theta band force directed simulations (Fig. 2b) further emphasise the difference between dystonia and controls, showing both more widespread engagement of the network and stronger communication between nodes in dystonia. At group level (Fig. 3) individuals with dystonia exhibited significantly increased 4–8 Hz GMNS values compared to healthy controls (Controls median 0.0018 IQR 0.0045, Dystonia median 0.0081 IQR 0.00147, Mann–Whitney U 31.5 p = 0.045).

### Global connectedness

**Figure 4:**
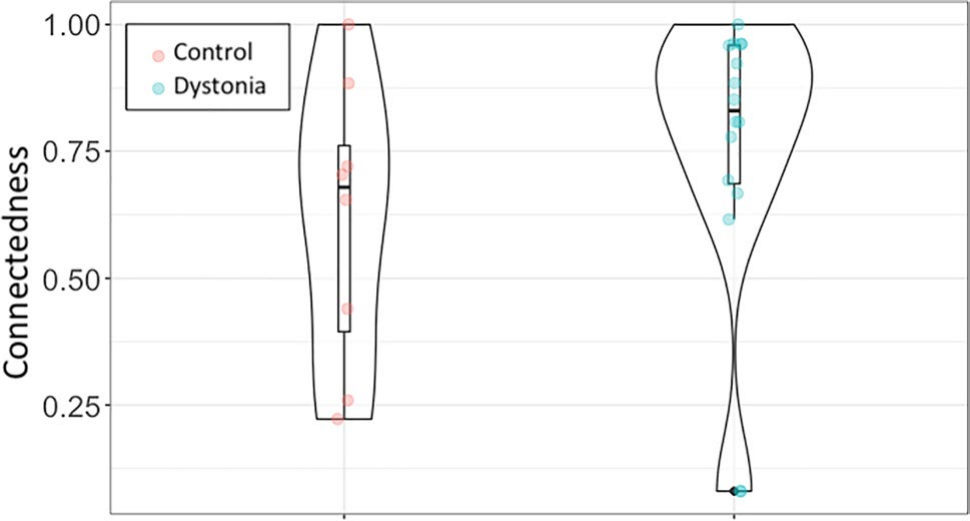
Global connectedness group differences. Violin plots with inverted kernel densities and data points. The patient group exhibits the tendency to “recruit” more EEG areas in response to SEP events, which is less consistent for the control group, but the difference is not statistically significant. Produced with R 3.6.0 and ggplot 3.3.2.

GC values for the Stretch EP related theta band network are shown in Fig. 4. Although connectedness was generally higher in dystonia than in controls, the difference in GC values was not significant at group level (Controls median 0.6788 IQR 0.5386, Dystonia median 0.8298 IQR 0.2877. Mann–Whitney U 43.5 p = 0.214).

### Assessment of potential confounding effects

#### Age

There was no correlation between age and GMNS (Spearman’s Rho − 0.217, p = 0.308) or between age and GC (Spearman’s Rho 0.024, p = 0.912).

#### Background EMG activity

Mean levels of rectified EMG were slightly higher in the dystonia group than the control group but the difference was not statistically significant (Extensor EMG controls median = 5.09 µV IQR 6.33; dystonia median = 8.24 µV IQR 13.48, Mann Whitney U = 44.0. p = 0.238; Flexor EMG controls median 4.48 µV IQR 6.21, dystonia median 9.19 µV IQR 13.54, Mann Whitney U = 36.0 p = 0.093).

#### DBS

The dystonia group comprised 11 patients without DBS and 5 patients with DBS. GMNS and GC values were comparable between those patients with and without DBS. (Median GMNS “With DBS” 0.0081, “without DBS” 0.0060, Mann Whitney U 25.5 p = 0.827; Median Connectedness “With DBS” 0.9232, “without DBS” 0.8077, Mann Whitney U 24.5 p = 0.743). The GMNS and GC analyses above were therefore performed on the dystonia group as a whole.

## Discussion

This novel study investigates sensorimotor neuronal network connectivity following a sensory stimulus and demonstrates significant abnormalities in network organisation in young people with dystonia compared with healthy controls. The Portable Hi5 device enables us to focus on responses to proprioceptive stimuli, which are thought to be of particular importance in dystonia, and the EEG methodology allows us to investigate specifically the dynamic changes in connectivity triggered by this stimulus. The current report demonstrates the feasibility of applying these methods in young people with movement disorders to investigate event-related network dynamics.

Preliminary findings from our novel application of these methods are:

1. Dynamic Global Microscale Nodal Strength (GMNS) is significantly stronger in dystonia than controls in the 4–8 Hz theta frequency band.
2. Individuals with dystonia tend to show more widespread engagement of different cortical regions in the activated network compared with controls.

### Dystonia as a network disorder

The concept of dystonia as a network disorder reconciles many observations and paradoxes. It accommodates the observations that: (a) dystonia can arise from brain lesions in many different neuroanatomical locations^1^ or indeed in individuals with no discernible structural lesion on imaging; (b) in animal studies, dystonic movements may be produced from manipulation of different brain regions^1^; (c) that there is often a delay between a brain lesion and the emergence of dystonia^19^, suggesting that maladaptive reorganisation in the network nodes or connections may be a key step in the development of dystonia.

The network model also accommodates the numerous pathophysiological mechanisms that have been demonstrated in dystonia and which may act diffusely across the sensorimotor network, such as reduced inhibition at multiple levels of the nervous system, including cortex, brainstem and spinal cord^8^, maladaptive plasticity^9,10,20^ and abnormal sensorimotor processing which has been demonstrated in multiple regions, including sensorimotor cortex^3,4^ and cerebellum^21^.

The observation of clear differences in pathophysiological findings between patients with different types of dystonia^16,19,22–24^ is also consistent with the network model. For example, in a large cohort of children with different types of dystonia, approximately 50% of those with acquired dystonia were found to have abnormalities of the N20 component of the Somatosensory Evoked Potential (SEP), while those with isolated idiopathic/genetic dystonias had normal SEPs^24^. This was interpreted in the context of the network model, with the authors pointing out that whilst individuals with idiopathic/genetic dystonias have sensory pathway abnormalities at the cortical processing level^3,8^, disruption of the sensory processing pathway at another node of the network (e.g. arrival of afferent information at the level of the primary sensory cortex) may contribute to the development of the motor disorder in children with acquired dystonia.

Evidence supporting the network model is provided by studies specifically investigating communication/connectivity between brain regions, often where no structural brain lesion can be identified. For example, abnormal connectivity in both the cortico-basal ganglia and the cortico-cerebellar networks has been demonstrated in writer’s cramp, both during “resting state” fMRI studies (functional connectivity)^5^ and during a motor task (effective connectivity)^6^. Writer’s cramp is typically associated with normal structural brain imaging, as is cervical dystonia. However, cervical dystonia can occasionally arise in the context of a focal brain lesion and a recent lesion network mapping study compared fMRI images from 25 previously published cases of “causal” cervical dystonia with resting state fMRI connectivity data from a large cohort of healthy controls^7^. Although patients showed brain lesions in multiple different regions, these were all demonstrated to fall within a single functionally connected brain network. The cerebellum and somatosensory cortex were the key regions showing abnormal connectivity in acquired cervical dystonia and the same regions were found to show abnormal connectivity in a cohort of patients with idiopathic cervical dystonia, highlighting a common network abnormality between idiopathic and acquired dystonias^7^.

Animal models of dystonia have also revealed abnormal connectivity between different regions of the sensorimotor network. For example DeSimone et al.^25^ demonstrated that a mouse model of DYT1 dystonia, which is typically associated with normal structural imaging, exhibited increased (resting state) functional connectivity across the striatum, thalamus and somatosensory cortex and reduced functional connectivity in the motor cortex and cerebellum compared with wild type mice. Interestingly, the same mouse model also shows impaired inhibitory modulation and plasticity of cortico-striatal synaptic transmission, suggesting there could be more than one mechanism at play^25^.

### EEG connectivity studies and low frequency oscillations

Connectivity investigations based on EEG methodologies, measuring cortical oscillatory coupling between regions of the sensorimotor network in specific frequency bands^26^ have also been used to demonstrate abnormal connectivity in dystonia^27–30^. There is clear evidence that frequency-specific neuronal oscillatory activity is important in a number of neurological disorders. In particular, low frequency (4–12 Hz) oscillatory activity is pathologically enhanced in the basal ganglia-cortical network in dystonia^11,31,32^ indicating abnormal network connectivity in the form of excessive synchronisation. Suppression of this enhanced pallidal low frequency activity by Deep Brain Stimulation is associated with clinical improvement^12^. Furthermore, Miocinovic et al. demonstrated that interhemispheric coherence between motor cortices was elevated in the alpha range during both rest and motor activity in patients with dystonia during a wash-out period after chronic DBS was turned off^29^.

### Interpretation and relevance of current findings

The current study in young people with dystonia both supports and extends these findings, demonstrating an exaggerated dynamic network response to a proprioceptive stimulus, which appears to generate theta-band synchronisation across the sensorimotor network that is excessive in both its distribution and in its magnitude (Figs. 2, 3). Our findings thus bring together the separate bodies of evidence described above in showing dystonia as a network disorder, characterised by low-frequency oscillatory activity and abnormal sensorimotor processing. The use of a stimulus-triggered (or event-related) approach allows the analysis to examine in particular the dynamic response to a sensory stimulus, i.e. within the microscale of time. Hsiao et al. found abnormal stimulus-triggered theta-band connectivity between the sensorimotor cortices in Paroxysmal Kinesigenic Dyskinesia^33^. However, to our knowledge, ours is the first study to investigate event-related dynamic connectivity changes in dystonia and adds further, complementary information to studies investigating resting state or context specific connectivity (e.g. during an on-going hand movement task).

The dynamic element of this study is important because this reflects the clinical picture: dystonic movements are often triggered by sensory stimuli or attempted voluntary movement. The study is also relevant in the context of how abnormal basal ganglia and cortical activity relates to manifestation of dystonic symptoms. Low frequency pallidal neuronal oscillations are coherent with dystonic EMG^12,13,34^. A number of genetic dystonias are characterised by the presence of abnormal low frequency intermuscular coherence^14,15^ and this observation has recently been extended to include acquired dystonias^16^, suggesting that a strong low frequency intermuscular coherence is a common feature across different types of dystonia. Interestingly, the magnitude of this exaggerated low frequency intermuscular coherence correlates with dystonia severity^16^ and is reduced by Deep Brain Stimulation^17^. In contrast there is evidence that the modulation of beta-range corticomuscular coherence by a proprioceptive stimulus shows different patterns of abnormality between patients with isolated genetic/idiopathic dystonia compared with acquired dystonia^16^. Understanding how abnormalities of sensorimotor processing in dystonia are translated into exaggerated or unwanted movements is key to developing and/or modifying therapies to maximise individualised patient benefit.

### Study limitations

The sample size in this study is small and the dystonia group is heterogeneous including patients both with and without DBS in situ. Larger studies will be required to confirm these preliminary findings. This study was initially designed to test the applicability of this analysis method to data recorded in an investigation of event-related changes in sensorimotor cortical EEG activity (which will be reported in full separately). However, despite the small sample size, a significant difference in GMNS between individuals with dystonia and controls was demonstrated, the physiological interpretation of which is in keeping with other findings in the literature. Moreover, the effect size is relatively large and will be informative for further studies of this kind. The findings in patients with and without DBS were similar and the presence of increased theta-band GMNS in patients both with and without DBS indicates that this finding could not be explained simply as an artefact relating to the DBS stimulus.

The dystonia group is also heterogeneous in terms of aetiology. Although this is to some extent a limitation, it is representative of the clinical context, and can also be considered a valuable aspect of the study. It is important to identify neurophysiological features which are common across different aetiological sub-types of dystonia, as these features may help us to understand both the underlying physiology of the disorder and the potential responsiveness of an individual to therapies such as neuromodulation. This study therefore paves the way for future work including larger numbers of subjects overall and in each aetiological sub-group.

The current analysis looks only at data recorded in response to right hand movement. Some of the subjects were left hand dominant (one control and three with dystonia) and it is possible that different patterns of dynamic connectivity are seen in response to movement of the dominant versus non-dominant hands. Future work will need to investigate and compare the connectivity responses to both hands.

Finally, this study looks only at global network connectivity measures, rather than specific topographical network connectivity, which would require larger group numbers. Future studies with larger sample size will address this.

## Conclusions

The current findings demonstrate significant microscale network organisation abnormalities in young people with dystonia. Specifically, the dynamic network response to a proprioceptive stimulus evokes excessive neuronal synchronisation in the theta band, engaging a higher number of cortical regions and with significantly stronger synchronisation than healthy controls. The observations are important in bringing together established findings of dystonia as a network disorder, characterised by pathologically enhanced low frequency neuronal oscillations and aberrant sensorimotor processing. Further studies are warranted to assess whether the dynamic network response shows common or different features across different types of dystonia.

## Materials and methods

### Subjects and experimental arrangement

The analyses were performed on data acquired in a larger study designed to investigate event-related changes in sensorimotor cortical EEG activity (which will be reported in full separately). The studies were performed on 16 young people with dystonia (8 female) aged 5–20 years, recruited from the Complex Motor Disorders Service clinic at Evelina London Children’s Hospital, and on 8 healthy controls (6 female) aged 6–19 years. Control subjects were recruited from local schools and clubs and from advertisement within the hospital. Only children with no history of neurological disorders were recruited as controls. Seven controls were right hand dominant by self (and parent) report. For patients, the diagnosis and classification of dystonia was confirmed by a consultant paediatric neurologist with specialist expertise in movement disorders (JPL). Details of the dystonia aetiology and phenotype are given in Table 1. Thirteen patients were right hand dominant by self (and parent) report. Five participants had bilateral Globus Pallidus internus DBS in situ. Severity of dystonia was assessed using the motor score of the Burke–Fahn–Marsden Dystonia Rating Scale (BFMDRS-m) and is included in Table 1 where available.

Subjects were seated comfortably at a table with their arm positioned in the armrest of a robotic wrist interface (named the “portable Hi5”), designed to produce controlled passive wrist extension movements. The Hi5 device was adapted from a previous study in which it was used for the investigation of tactile sensation in adults^35^ and is in keeping with a similar device used for the investigation of brain development in neonates^36^, but was modified to accommodate the current protocol (Fig. 1A,B). The portable Hi5 interface is designed so that it can be easily mounted on a table. The forearm is positioned and supported in mid-supination, parallel to the table, while the hand is strapped to a customised hand-piece with the wrist joint aligned with the pivot point. Two mirror-image hand-pieces were designed to suit the hand of the children in the age-range of the study and could be interchanged to allow movements to be performed for either the right or left hand. The hand-piece was moved ad hoc by a motor to produce a brief passive wrist extension, followed by return to neutral, thus providing a brief stretch of the wrist flexors. The movement followed the profile of the half period of a sine wave (1 Hz), with rise time of 240 ms and a target of 12° from the neutral position. The actual excursion of the wrist was in the range 5–15° due to intrinsic mechanical factors and varying resistance of the subject’s hand (Fig. 1C—movement profile). Stimulus control, monitoring and synchronization between stimuli and EEG recordings was achieved through a custom code developed by the authors in the LabVIEW software environment (National Instruments, Austin, TX, USA).

### Recordings

Scalp EEG was recorded with Ag/AgCl electrodes sited according to the 10–20 international system at Fp1, Fp2, F7, F3, Fz, F4, F8, Fc5, FC1, FcZ, FC2, FC6, T3/C5, C3, Cz, C4, T4/C6, CP5, CP1, CP2, CP6, P7/T5, P3, Pz, P4, P8/T6, Oz with the ground electrode at AFz. Electrodes were applied to the scalp using conductive paste (Ten20, Weaver and Company) and impedances were maintained below 10 kOhm. Surface EMG was recorded using self-adhesive electrodes (Neuroline 700, Ambu) applied to forearm flexors and forearm extensors in a belly-tendon montage. EEG and EMG were amplified, filtered between DC and 500 Hz and sampled at 2500 Hz using a BrainVision system (BrainAmp MR Plus).

The Hi5 device was set to repeat the wrist extension movement at pseudorandom intervals of  5 s ± 0.5 s to prevent anticipation. Movements were performed in blocks of 10 and the participant was given the opportunity to rest between blocks if required. Up to 160 wrist extension movements were recorded for each hand. The movement of the hand-piece was measured during each experiment via a position sensor incorporated into the device and was recorded at 1000 Hz. Additionally, the LabVIEW environment provided visual feedback so that the researcher could monitor the hand position throughout the experiment. A TTL (transistor-transistor logic) pulse was passed from the Hi5 interface to the EEG recording system to synchronise the movement onset with the simultaneous EEG and EMG recordings. An additional movement sensor was applied to the wrist joint.

###  Offline analysis

The profile of the wrist movements was analysed in Matlab. Epochs with an abnormal wrist movement profile (i.e. with excursion of < 5° or > 15° or with excessive movement during the resting period) were excluded. A lag time of 3–30 ms was observed between the TTL pulse and the actual movement onset, reflecting slight differences in inertia of the wrist joint between subjects. To account for this, the true movement onset was defined as the sample point at which a continuous increase in joint position, as recorded by the Hi5 interface, was observed over 5 consecutive sample points. The EEG markers based on the TTL pulse were then amended to reflect this true movement onset for each epoch, using the Brain Vision Edit Markers function.

EEG data were pre-processed in BrainVision Analyser Version 2.2 (https://www.brainproducts.com). Recordings were down-sampled to 1000 Hz (to match position data sampling) and movement onset markers were edited as above. A high-cut filter of 70 Hz was applied offline (in all subjects) to avoid contamination of recordings by DBS artefact. A 50 Hz notch filter was also applied to reduce contamination by mains artefact. Data were then segmented into epochs based on the corrected movement onset markers. Artefact rejection was performed manually, to remove those epochs with inadequate wrist movement profile and those contaminated by excessive movement or eye blink artefacts. Remaining epochs were then averaged to reveal the Evoked Potential/Event related Potential (Fig. 1d) for each hand in each subject. For the purpose of this feasibility study, data for the right hand only are reported. EEG data were exported for further analysis using Matlab software customised by the authors^37^. EMG data were processed in BrainVision Analyser. The mean amplitude of rectified EMG for each muscle was calculated in each subject to allow comparison of background muscle activity between groups.

### Event related network dynamics

To estimate time–frequency coupling interactions with high resolution, here we make use of the Wavelet Transform Coherency (WTC). WTC exhibits better frequency and time resolution compared to short time Fourier Transform and other methods used commonly in EEG analysis^38^, at the cost of higher processing load. Specifically, WTC measures the correlation between two signals based on their wavelet transforms. Given two ${x}_{i}\left(t\right)$ and ${x}_{j}\left(t\right)$ processes and their wavelet transforms ${w}_{i}\left(\sigma ,\tau \right)$ and ${w}_{j}\left(\sigma ,\tau \right)$, the wavelet cross-spectrum is defined as

1 $${W}_{ij}\left(\sigma ,\tau \right)=S\left({w}_{i}^{*}\left(\sigma ,\tau \right){w}_{j}\left(\sigma ,\tau \right)\right)$$

where *S* is the smoothing operator in time ${s}_{\tau }$ and scale ${s}_{\sigma }$ and in which, in order to provide a constant variance for all scales, its averaging kernel operates according to the reproducing kernel^39^. Consequently, the smoothed time–frequency wavelet coherency is defined as

2 $${R}_{ij}\left(\sigma ,\tau \right)=\frac{{W}_{ij}\left(\sigma ,\tau \right)}{{\left(S\left({\left|{w}_{ij}\left(\sigma ,\tau \right)\right|}^{2}\right) \cdot S\left({\left|{w}_{j}\left(\sigma ,\tau \right)\right|}^{2}\right)\right)}^{1/2}}$$

To allow the quantification of causal dependence between two time-series in terms of phase, we make use of a Morlet wavelet^40^ as the mother wavelet defined as

3 $$\psi \left(\tau \right)={\pi }^{-\frac{1}{4}}{e}^{i{\omega }_{0}\tau }{e}^{\frac{{-\tau }^{2}}{2}}$$

where ${\omega }_{0}=2\pi {f}_{0}$ is the non-dimensional frequency. The spread of the wavelet’s energy in time and frequency determines the minimum and maximum scales^41^, here taken to be ${\omega }_{0}=6$ to satisfy the admissibility condition^42^ and 12 voices per octave.

### Ensemble wavelet transform coherency

To estimate connectivity values across multiple time-locked repetitive Stretch EP events we performed ensemble calculation of WTC i.e. estimated WTC, by taking into account all of the available *n* epochs (Eq. 4) within every subject.

4 $${\widehat{R}}_{ij}\left(\sigma ,\tau \right)=\frac{\sum_{i=1}^{n}{W}_{ij}\left(\sigma ,\tau \right)}{{\left(\sum_{n=1}^{N}S\left({\left|{w}_{i}\left(\sigma ,\tau \right)\right|}^{2}\right)\cdot \sum_{n=1}^{N}S\left({\left|{w}_{j}\left(\sigma ,\tau \right)\right|}^{2}\right)\right)}^{1/2}}$$

where $n=1,\dots ,N$ is the number of event epochs. In principle, when a number of repetitive realisations of the same random process is present it is sufficient to rely on ergodicity, with stationarity no longer being a prerequisite to estimate coherency^37,43^.

### The imaginary part of wavelet transform coherency

In EEG, a single source of activity can contribute to measurements in adjacent electrodes, usually called “volume conduction”. To rule out such spurious connectivity measurements and in order to interpret WTC as a measure that reflects interaction between areas we only take into account the Imaginary part of WTC^44^.

5 $$IWTC=Imag\left({\widehat{R}}_{ij}\left(\sigma ,\tau \right)\right)$$

### Significance testing against random coherency

Additional to ensemble WTC, to test for significance against random coupling between brain areas we apply non-parametric bootstrapping by constructing surrogate data of randomly chosen background EEG epochs under the null hypothesis of independence^45^. Subject-specific significant connectivity values were estimated using a *α* = 0.05 level of significance with an approximation of Z = 100 number of bootstrap resampled estimates of the background epochs. The typical value of Z repetitions is between 25 and 100^46^. Specifically for a κ background epoch and relevant frequency data of a pair of electrodes, *Si(f ), Sj(f )*, random samples Si*(f ), Sj*(f ) were drawn using a pseudo-random number generator with replacement from *Si(f)* = *{di(f,1),…,di(f,n)}* and *Sj(f)* = *{dj(f,1), …,dj(f,n)}* of *n* periodograms over *K* background trials in a 1-by-1 scheme in order to populate the model with K-resampled trials. $Z\cdot {IWTC}_{ij}^{*}(f)$ bootstrap statistics derive from a Z number of repetitions of the above procedure. For the confidence interval estimation of a given level of significance, where

$$P(IWTClower ({|IWTCij(\omega )|}^{2}(\le {|IWTCij(\omega )|}^{2}\le IWTCupper({|IWTCij(\omega )|}^{2}) = 1 - a,$$

the percentiles of the ordered distribution of all bootstrap estimates were calculated^47^. Subsequently, lengths exceeding a certain quantile of the length distribution then signifies intrinsic coherency for the two processes^39^.

### Global microscale nodal strength

To estimate the overall engagement of areas into the short-lived networks related to the Stretch EP, we calculated the median of weighted degrees across all nodes, defined here as Global Microscale Nodal Strength (GMNS).

We define as weighted degree the sum of connectivity values *w* of a node *i* with every other *j* node, against the number of all nodes *N*

6 $${d}_{i}=\frac{1}{N}{\sum }_{j\in N}{a}_{ij}$$

where *aij* = connectivity value for the i-by-j pair of nodes/electrode areas, *N* = total number of nodes.

### Global connectedness

To estimate the spatial extent of the Stretch EP networks, we calculated Global Connectedness (GC) for each subject as a measure of the EEG areas those engage. In technical terms, GC is the density of the binary adjacency connectivity matrix which was calculated as the ratio of EEG areas with non-zero connectivity values (${d}_{i}>0$) per all available EEG areas.

### Statistics

GMNS and GC values were compared between groups. Only significant values of time–frequency connectivity were considered for the GMNS and GC calculation of each subject. Statistical analyses were performed using SPSS (Version 25) and R (Version 3.6). Normality tests for each group were conducted by visual inspection of quantile–quantile plots and Shapiro–Wilk’s tests, and non-parametric tests were used where the distribution was significantly different from normal. Group comparisons were made using the independent samples t test or Mann–Whitney U test, as appropriate, using a two-tailed analysis. Spearman’s correlation was used for correlation analysis, testing for age effects.

### Ethical approval

Ethical approval was obtained from the London-Harrow National Research Ethics Committee, London, UK (17/LO/0439). Informed written consent was obtained from the participant or, if under 16 years old, from parents with assent from the child. The studies were conducted in accordance with the declaration of Helsinki.

## Data Availability

The data that support the findings of this study will be available in the KCL data repository after completion of the full project. The data management plan approved by the funders states the following: ***The study team’s exclusive use of the data*** *The research team will have exclusive use of the data for the project duration and three years afterwards to allow for publications to be achieved. After this period, the majority of data would be made available for data sharing *via* the King’s College London data repository.*

## References

[1] Neychev VK, Gross RE, Lehericy S, Hess EJ, Jinnah HA (2011). The functional neuroanatomy of dystonia. Neurobiol. Dis..

[2] Nelson AJ, Blake DT, Chen R (2009). Digit-specific aberrations in the primary somatosensory cortex in Writer's cramp. Ann. Neurol..

[3] Tinazzi M (2000). Abnormal central integration of a dual somatosensory input in dystonia. Evidence for sensory overflow. Brain.

[4] Frasson E (2001). Somatosensory disinhibition in dystonia. Mov. Disord..

[5] Dresel C (2014). Multiple changes of functional connectivity between sensorimotor areas in focal hand dystonia. J. Neurol. Neurosurg. Psychiatry.

[6] Rothkirch I (2018). Dynamic causal modeling revealed dysfunctional effective connectivity in both, the cortico-basal-ganglia and the cerebello-cortical motor network in writers' cramp. NeuroImage Clin..

[7] Corp DT (2019). Network localization of cervical dystonia based on causal brain lesions. Brain.

[8] Hallett M (2011). Neurophysiology of dystonia: The role of inhibition. Neurobiol. Dis..

[9] Quartarone A (2008). Abnormal plasticity of sensorimotor circuits extends beyond the affected body part in focal dystonia. J. Neurol. Neurosurg. Psychiatry.

[10] Quartarone A, Pisani A (2011). Abnormal plasticity in dystonia: Disruption of synaptic homeostasis. Neurobiol. Dis..

[11] Neumann WJ (2015). Cortico-pallidal oscillatory connectivity in patients with dystonia. Brain.

[12] Barow E (2014). Deep brain stimulation suppresses pallidal low frequency activity in patients with phasic dystonic movements. Brain.

[13] Sharott A (2008). Is the synchronization between pallidal and muscle activity in primary dystonia due to peripheral afferance or a motor drive?. Brain.

[14] Foncke EM, Bour LJ, van der Meer JN, Koelman JH, Tijssen MA (2007). Abnormal low frequency drive in myoclonus-dystonia patients correlates with presence of dystonia. Mov. Disord..

[15] Grosse P (2004). Patterns of EMG-EMG coherence in limb dystonia. Mov. Disord..

[16] McClelland VM, Cvetkovic Z, Lin JP, Mills KR, Brown P (2020). Abnormal patterns of corticomuscular and intermuscular coherence in childhood dystonia. Clin. Neurophysiol..

[17] Doldersum E (2019). Intermuscular coherence as biomarker for pallidal deep brain stimulation efficacy in dystonia. Clin. Neurophysiol..

[18] Campfens SF, Meskers CG, Schouten AC, van Putten MJ, van der Kooij H (2015). Stretch evoked potentials in healthy subjects and after stroke: A potential measure for proprioceptive sensorimotor function. IEEE Trans. Neural. Syst. Rehabil. Eng..

[19] Trompetto C (2012). Corticospinal excitability in patients with secondary dystonia due to focal lesions of the basal ganglia and thalamus. Clin. Neurophysiol..

[20] Tamura Y (2009). Disordered plasticity in the primary somatosensory cortex in focal hand dystonia. Brain.

[21] Popa T (2018). Abnormal cerebellar processing of the neck proprioceptive information drives dysfunctions in cervical dystonia. Sci. Rep..

[22] Kojovic M (2013). Secondary and primary dystonia: Pathophysiological differences. Brain.

[23] McClelland VM (2016). Differences in globus pallidus neuronal firing rates and patterns relate to different disease biology in children with dystonia. J. Neurol. Neurosurg. Psychiatry.

[24] McClelland VM (2018). Somatosensory evoked potentials and central motor conduction times in children with dystonia and their correlation with outcomes from deep brain stimulation of the Globus pallidus internus. Clin. Neurophysiol..

[25] DeSimone JC (2016). In vivo imaging reveals impaired connectivity across cortical and subcortical networks in a mouse model of DYT1 dystonia. Neurobiol. Dis..

[26] Herz DM (2012). Task-specific modulation of effective connectivity during two simple unimanual motor tasks: A 122-channel EEG study. Neuroimage.

[27] Jin SH, Lin P, Auh S, Hallett M (2011). Abnormal functional connectivity in focal hand dystonia: Mutual information analysis in EEG. Mov. Disord..

[28] Jin SH, Lin P, Hallett M (2011). Abnormal reorganization of functional cortical small-world networks in focal hand dystonia. PLoS One.

[29] Miocinovic S, Miller A, Swann NC, Ostrem JL, Starr PA (2017). Chronic deep brain stimulation normalizes scalp EEG activity in isolated dystonia. Clin. Neurophysiol..

[30] Melgari JM (2013). Movement-induced uncoupling of primary sensory and motor areas in focal task-specific hand dystonia. Neuroscience.

[31] Silberstein P (2003). Patterning of globus pallidus local field potentials differs between Parkinson's disease and dystonia. Brain.

[32] Liu X (2008). The sensory and motor representation of synchronized oscillations in the globus pallidus in patients with primary dystonia. Brain.

[33] Hsiao FJ, Hsu WY, Chen WT, Chen RS, Lin YY (2016). Abnormal somatosensory synchronization in patients with paroxysmal kinesigenic dyskinesia: A magnetoencephalographic study. Clin. EEG Neurosci..

[34] Moll CK (2014). Asymmetric pallidal neuronal activity in patients with cervical dystonia. Front. Syst. Neurosci..

[35] Wilhelm E (2016). Investigation tactile sensation in the hand using a robot-based tactile assessment tool. EuroHaptics.

[36] Allievi AG, Melendez-Calderon A, Arichi T, Edwards AD, Burdet E (2013). An fMRI compatible wrist robotic interface to study brain development in neonates. Ann. Biomed. Eng..

[37] Sakellariou D, Koupparis AM, Kokkinos V, Koutroumanidis M, Kostopoulos GK (2016). Connectivity measures in EEG microstructural sleep elements. Front. Neuroinform..

[38] Zhan Y, Halliday D, Jiang P, Liu X, Feng J (2006). Detecting time-dependent coherence between non-stationary electrophysiological signals—a combined statistical and time-frequency approach. J. Neurosci. Methods.

[39] Maraun D, Kurths J, Holschneider M (2007). Nonstationary Gaussian processes in wavelet domain: Synthesis, estimation and significance testing. Phys. Rev. E.

[40] Kronland-Martinet R, Morlet J, Grossman A (1987). Analsyis of sound patterns through wavelet transforms. Int. J. Pattern Recogn..

[41] Lilly JM, Olhede SC (2009). Higher-order properties of analytic wavelets. IEEE Trans. Signal Process..

[42] Farge M (1992). Wavelet transforms and their applications to turbulence. Annu. Rev. Fluid Mech..

[43] Klein A, Sauer T, Jedynak A, Skrandies W (2006). Conventional and wavelet coherence applied to sensory-evoked electrical brain activity. IEEE Trans. Biomed. Eng..

[44] Nolte G (2004). Identifying true brain interaction from EEG data using the imaginary part of coherency. Clin. Neurophysiol..

[45] Schreiber T, Schmitz A (2000). Surrogate time series. Phys. D.

[46] Efron, B. & Tibshirani, R. J. *An Introduction to the Bootstrap*. (1994).

[47] Zoubir, A. M. in *Proceedings (ICASSP ’05) IEEE International conference on Acoustics, Speech, and Signal Processing.*

[48] Fruchterman TMJ, Reingold EM (1991). Graph drawing by force-directed placement. Softw. Pract. Exp..

